# Synthesis and Bioactivities of Novel Pyrazole Oxime Derivatives Containing a 5-Trifluoromethylpyridyl Moiety

**DOI:** 10.3390/molecules21030276

**Published:** 2016-02-27

**Authors:** Hong Dai, Jia Chen, Hong Li, Baojiang Dai, Haibing He, Yuan Fang, Yujun Shi

**Affiliations:** 1 College of Chemistry and Chemical Engineering, Nantong University, Nantong 226019, China; daihong_2015@aliyun.com (H.D.); 15642891665@163.com (J.C.); fyuan6586@aliyun.com (Y.F.); 2 Nantong Fengtian Chemical Industry Co. Ltd., Nantong 226005, China; leehomer@sina.com; 3 School of Chemical and Biological Engineering, Nantong Vocational University, Nantong 226007, China; dbj20110802@163.com

**Keywords:** pyrazole oxime, 5-trifluoromethylpyridyl, synthesis, bioactivity

## Abstract

In this study, in order to find novel biologically active pyrazole oxime compounds, a series of pyrazole oxime derivatives containing a 5-trifluoromethylpyridyl moiety were synthesized. Preliminary bioassays indicated that most title compounds were found to display good to excellent acaricidal activity against *Tetranychus cinnabarinus* at a concentration of 200 μg/mL, and some designed compounds still showed excellent acaricidal activity against *Tetranychus cinnabarinus* at the concentration of 10 μg/mL, especially since the inhibition rates of compounds **8e**, **8f**, **8l**, **8m**, **8n**, **8p**, and **8q** were all 100.00%. Interestingly, some target compounds exhibited moderate to good insecticidal activities against *Plutella xylostella* and *Aphis craccivora* at a concentration of 200 μg/mL; furthermore, compounds **8e** and **8l** possessed outstanding insecticidal activities against *Plutella xylostella* under the concentration of 50 μg/mL.

## 1. Introduction

**Figure 1:**
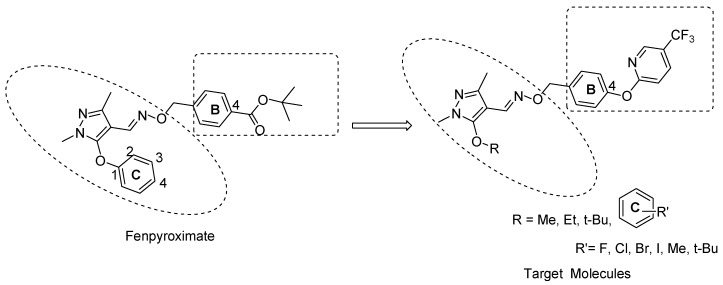
The design of the target molecules.

Agrochemicals, together with genetically modified insect-resistant crops and biological plant protection methods, prevent severe harvest losses caused by phytophagous insects and mites [1,2,3]. Over the past decades, a great variety of chemical pesticides have been developed and used in the protection of crops, among which pyrazole derivatives occupy a considerable proportion in insecticides, germicides and acaricides [4,5,6,7]. As a well-known pyrazole-based pesticide developed by Nihon Nohyaku Co. in 1991, Fenpyroximate (Figure 1) has been widely used in protecting various crops due to its high efficiency against agricultural mites such as *Polyphagotarsonemus latus* Banks and *Tetranychus urticae* Koch, and its low toxicity to mammals [8]. Unfortunately, continuous application of Fenpyroximate in recent years led to the occurrence of resistance from some field populations of *T. urticae* [9]. Researchers are therefore propelled to search for new compounds that are highly active against phytophagous mites, and the development of novel analogues of Fenpyroximate is extraordinarily focused on [10,11,12].

Fenpyroximate is structurally characterized by the unique 4-pyrazole oxime, which is recognized as an irreplaceable pharmacophore for acaricidal or insecticidal activities. In the process of developing new Fenpyroximate derivatives, modification of phenyl B (Figure 1) and its substituents was mainly concentrated on with the 4-pyrazole oxime moiety retained. For example, Dai and coworkers reported some acaricidal or insecticidal Fenpyroximate mimics that were obtained by replacing the phenyl B (Figure 1) with thiazole [13], or a pyridyl group [14]. Many of these efforts succeeded in getting some heterocycle-based pyrazole oxime compounds that displayed promising biological activities. Therefore, structural optimization of the phenyl B of Fenpyroximate (Figure 1) to explore novel bioactive molecules is a reasonable design.

As we know, due to its special aromaticity, basicity and hydrophilicity, a pyridyl group is a very commonly used active moiety in pesticidal molecules [15,16,17,18,19,20,21]. In addition, many investigations have indicated that introducing CF_3_ into heterocyclic molecules mostly results in the improvement of physical, chemical and biological properties [22,23]. In fact, it has been proved that CF_3_-substituted pyridine is a highly efficient functional group in active pesticidal molecules, for instance neonicotinoids insecticide Sulfoxaflor [24], and dichloropropene insecticide Pyridalyl [25], each of which has a similar trifluoromethyl pyridyl fragment.

Encouraged by these reports, in this study we integrated the 5-trifluoromethyl pyridyl unit into the scaffold of Fenpyroximate for the first time, and the substituents on the 5-position of the pyrazole ring are screened and optimized (Figure 1). Insecticidal and acaricidal activities of the target compounds were tested accordingly.

## 2. Results and Discussion

### 2.1. Chemistry

**Scheme 1:**
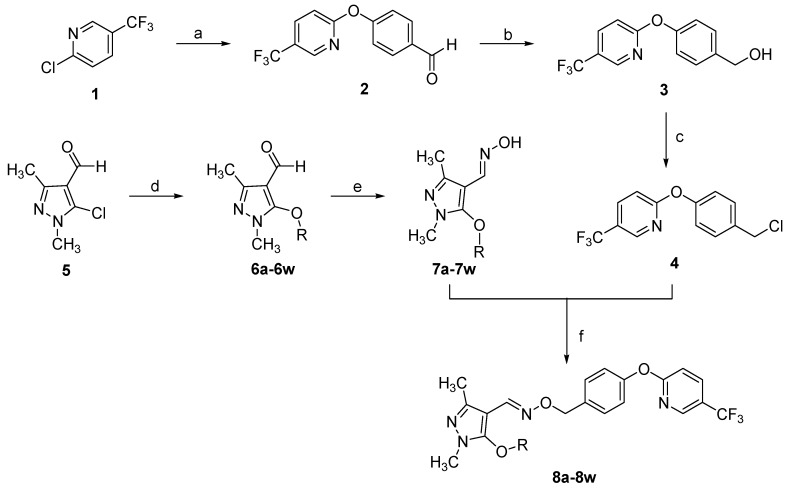
Synthesis of the title compounds **8a**–**8w**. *Reagents and conditions:* (**a**) 4-hydroxybenzaldehyde, Cs_2_CO_3_, *N*,*N*-dimethylformamide, 105 °C for 10 h, 75% for **2**; (**b**) NaBH_4_, ethanol, 0 °C for 3 h, 90% for **3**; (**c**) thionyl chloride, *N*,*N*-dimethylformamide, dichloromethane, r.t. for 8 h, 82% for **4**; (**d**) NaOR (R = Me, Et and *t*-Bu), ROH, 30 °C for 3 h, 45 °C for 2 h, 41%–49% for **6a**–**6c**; NaOR (R = substituted phenyl), dimethylsulfoxide, 105 °C for 4–15 h, 60%–81% for **6d**–**6w**; (**e**) hydroxylamine hydrochloride, potassium hydroxide, methanol or ethanol, reflux for 6–16 h, 65%–87% for **7a**–**7w**; (**f**) compound **4**, Cs_2_CO_3_, acetonitrile, reflux for 10–18 h, 44%–63% for **8a**–**8w**.

As shown in Scheme 1, 23 pyrazole oxime derivatives bearing a 5-trifluoromethyl pyridyl moiety were conveniently synthesized. The synthon **4** was synthesized from commercially available 2-chloro-5-trifluoromethylpyridine (**1**) as shown in Scheme 1. Compound **1** reacted with 4-hydroxybenzaldehyde using Cs_2_CO_3_ as the base and *N*,*N*-dimethylformamide as the solvent to give intermediate **2** in 75% yield. The next reaction with NaBH_4_ afforded compound **3** in 90% yield. Further chlorination of compound **3** obtained intermediate **4** in satisfactory yield. The addition of a few drops of *N*,*N*-dimethylformamide accelerated the chlorination reaction. The pyrazole oximes **7a**–**7w** were prepared via two steps from pyrazole carbaldehyde **5**. Benefitting from the activated C-Cl bond by the electron-withdrawing formyl group at the 4-position of the pyrazole ring, the introduction of either alkyl alcohol or substituted phenol nucleophiles into 5-chloro-1,3-dimethyl-1*H*-pyrazole-4-carbaldehyde (**5**) was performed smoothly and successfully, and afforded 5-substituted pyrazole-4-carbaldehydes (**6a**–**6w**). The condensation of intermediates **4** and **7a**–**7w** proceeded smoothly with Cs_2_CO_3_ as the base and acetonitrile as the solvent to provide the title compounds **8a**–**8w** in good yields. The structures of all the target compounds were analyzed and confirmed by ^1^H-NMR, ^13^C-NMR and elemental analyses.

### 2.2. Biological Activities

#### 2.2.1. Acaricidal Activity

The acaricidal activity of all the title compounds against *Tetranychus cinnabarinus* was evaluated and the data are listed in Table 1. The results indicated that 5-alkoxy pyrazole derivatives **8a**, **8b** and **8c** possessed no acaricidal activity at a concentration of 200 μg/mL. For the other 5-aryloxy-substituted compounds **8d**–**8w**, an obvious substituent effect was found on the phenyl ring C. When the substituent at the 2-position of phenyl C (Figure 1) was halogen (**8d**, **8g** and **8j**) or methoxy (**8o**), it would reduce the acaricidal activity, and the mortality obviously declined depending on the concentration and disappeared at the concentration of 10 μg/mL. Moreover, 3-substituted phenyl C (Figure 1) affected the acaricidal activities in a similar manner. The introduction of halogen atoms (**8h** and **8k**) led to the loss of acaricidal activity, except for that of 3-fluoro derivative (**8e**). As well as unsubstituted compound **8n**, compound **8e** retained 100.00% mortality, even at 10 μg/mL. The results seem to show that compared with the inductive effect, steric hindrance of the 2- or 3-position on phenyl C (Figure 1) plays a more important role in regulating the acaricidal activity. In contrast, it exhibited a good tolerance of 4-substituents on phenyl C (Figure 1 and Table 1), because the introduction of halogen atoms (**8f**, **8l** and **8m**), methoxy (**8p**), methyl (**8q**) or trifluoromethoxy (**8s**) did not affect the acaricidal activities at concentrations ranging from 200 μg/mL to 10 μg/mL. However, 4-*tert* butyl compound **8r** was an exception, whose activity against *T. cinnabarinus* disappeared completely at a concentration of 200 μg/mL. Additionally, among disubstituted derivatives, compounds **8t** and **8w** displayed relatively higher acaricidal activity than compounds **8u** and **8v** from the concentrations of 200 μg/mL to 50 μg/mL.

#### 2.2.2. Insecticidal Activities

Besides acaricidal potencies, the insecticidal activities of the new compounds were also explored on *Plutella xylostella* and *Aphis craccivora*. As shown in Table 2, some of the obtained compounds displayed good insecticidal activities against *P. xylostella*. For instance, the mortalities of compounds **8e**, **8f**, **8i**, **8l**, **8m**, **8n**, **8p**, and **8q** against *P. xylostella* were all 100.00% at 200 μg/mL. Moreover, some of them showed good insecticidal activities against *P. xylostella* when the concentration was reduced to 50 μg/mL; compounds **8e** and **8l** possessed relatively higher insecticidal activities against *P. xylostella* than other derivatives. Some target compounds also demonstrated moderate to good insecticidal activities against *A. craccivora* at 200 μg/mL; for example, compounds **8e**, **8h**, **8i**, and **8r** had 100.00%, 85.33%, 95.28%, and 100.00% inhibition rates, respectively. In fact, similar to that of acaricidal activities, the compounds possessing more potent insecticidal abilities against *P. xylostella* were 4-substituted derivatives with compound **8e** as the only exception. When it comes to *A. craccivora*, the structure-activity relationships were not obvious. Overall, they were more potent against *P. xylostella* than against *A. craccivora*.

All the above data implied that structural modification of Fenpyroximate by a 5-trifluoromethyl pyridyl unit could produce some new compounds with good biological activities. To get more active derivatives, further analogue synthesis and structural optimization are well under way.

## 3. Experimental Section

### 3.1. Chemistry

#### 3.1.1. General Procedures

All reagents were chemically pure and solvents were dried according to standard methods. The ^1^H-NMR and ^13^C-NMR spectra were obtained on a Bruker AV400 spectrometer (400 MHz, ^1^H; 100 MHz, ^13^C, Bruker, Billerica, MA, USA) in CDCl_3_ with tetramethylsilane as the internal standard. The melting points were determined on an X-4 binocular microscope melting point apparatus (Beijing Tech Instrument Co., Beijing, China) and are uncorrected. Elemental analyses were determined on a Yanaco CHN Corder MT-3 elemental analyzer (Yanaco, Kyoto, Japan). The reactions were monitored by analytical thin-layer chromatography (TLC) with ultraviolet (UV) light and TLC was carried out on silica gel GF_254_. The intermediates 5-chloropyrazole aldehyde **5** and 5-alkoxy pyrazole aldehyde **6a**–**6c** were synthesized according to the reported procedures [26]. The 5-Substituted pyrazole oximes **7a**–**7w** were prepared by the literature method [11].

#### 3.1.2. Synthesis of 4-(5-Trifluoromethylpyridin-2-yloxy)benzaldehyde (**2**)

To a solution of 4-hydroxybenzaldehyde (6.4 g, 52.5 mmol) in *N*,*N*-dimethylformamide (150 mL) was added Cs_2_CO_3_ (16.3 g, 50 mmol), the mixture was then stirred for 20 min at room temperature, followed by adding compound **1** (9.1 g, 50 mmol). The resulting mixture was then heated slowly to 105 °C and stirred for 10 h. After cooled to room temperature, the solvent was evaporated *in vacuo*. The slurry was then distributed in water (150 mL) and ethyl acetate (100 mL), and the separated water phase was then extracted with ethyl acetate (3 × 50 mL). The combined organic layer was washed by water and brine, dried over anhydrous Na_2_SO_4_, and concentrated in rotatory evaporator to afford compound **2** in 75% yield as a white solid, which was used for the following transformations without further purification.

#### 3.1.3. Synthesis of 4-(5-Trifluoromethylpyridin-2-yloxy)phenylmethanol (**3**)

Intermediate **2** (13.4 g, 50 mmol) was dissolved in ethanol (100 mL) and cooled to 0 °C. To the solution was added NaBH_4_ (3.8 g, 100 mmol) in portions over 30 min. After being stirred at 0 °C for 3 h, the reaction mixture was poured into water, followed by adding 5% hydrochloric acid to adjust pH to 5–6. The resulting solution was extracted by chloroform (3 × 50 mL), dried over anhydrous Na_2_SO_4_ and concentrated under reduced pressure to give compound **3** in 90% yield, which was used in the next procedure without further purification.

#### 3.1.4. Synthesis of 2-(4-Chloromethylphenoxy)-5-(trifluoromethyl)pyridine (**4**)

A solution of intermediate **3** (13.5 g, 50 mmol) in dichloromethane (80 mL) was cooled in ice-water bath followed by adding thionyl chloride (8.9 g, 74.8 mmol) dropwise over 20 min. Then a few drops of *N*,*N*-dimethylformamide was added thereto. The resulting solution was stirred at room temperature for 8 h. The reaction mixture was quenched by trash ice, then the organic phase was separated, washed by water and saturated NaHCO_3_, dried over anhydrous Na_2_SO_4_, and evaporated *in vacuo* to produce compound **4** (yield 82%) as a white solid; ^1^H-NMR (CDCl_3_): δ 8.36 (s, 1H, Py-H), 7.84 (dd, *J*_1_ = 8.8 Hz, *J*_2_ = 2.4 Hz, 1H, Py-H), 7.38 (d, *J* = 8.4 Hz, 2H, Ar-H), 7.07 (d, *J* = 8.4 Hz, 2H, Ar-H), 6.96 (d, *J* = 8.8 Hz, 1H, Py-H), 4.54 (s, 2H, CH_2_). Anal. Calcd for C_13_H_9_ClF_3_NO: C 54.28; H 3.15; N 4.87. Found: C 54.41; H 3.03; N 4.70.

#### 3.1.5. General Procedure for the Preparation of **6d**–**6w**

To a solution of substituted phenol (26 mmol) in absolute ethanol (50 mL) was added sodium hydroxide (26 mmol) at room temperature. The mixture was heated to reflux for 3–5 h. After the removal of the solvent, the residue was dissolved in dimethylsulfoxide (50 mL), to the resulting mixture was added 5-chloro-1,3-dimethyl-1*H*-pyrazole-4-carbaldehyde (**5**) (20 mmol) in portions. Then the solution was heated to 105 °C and maintained at that temperature for 4–15 h and cooled to room temperature. The reaction mixture was poured into water (100 mL) and extracted with ethyl acetate (3 × 50 mL). The organic layer was washed with water (3 × 25 mL) and dried over anhydrous Na_2_SO_4_, filtered and evaporated to produce the corresponding carbaldehydes **6d**–**6w**, with yields ranging from 60% to 81% [11].

#### 3.1.6. General Procedure for the Preparation of **8a**–**8w**

To a stirred solution of intermediate **4** (7.2 mmol), compound **7** (6 mmol) in anhydrous acetonitrile (30 mL) was added Cs_2_CO_3_ (7.2 mmol) at room temperature, the resulting mixture was heated to reflux for 10–18 h. After cooled to room temperature, the reaction mixture was filtered. After most of the solvent had been evaporated under reduced pressure, the residue was admixed with water (100 mL) and extracted with dichloromethane (3 × 30 mL). The combined organic layer was washed with water (3 × 30 mL), and dried over anhydrous Na_2_SO_4_. The solvent was removed using a rotary evaporator to give a residue, which was then separated by silica gel column chromatography using petroleum ether and ethyl acetate (*v*/*v* = 30:1) as eluent to afford the target compounds **8a**–**8w**, with yields ranging from 44% to 63%. All 23 pyrazole oxime derivatives **8a**–**8w** were novel and the physical and spectral data for these compounds are listed below.

*1,3-Dimethyl-5-methyloxy-1H-pyrazole-4-carbaldehyde-O-[4-(5-trifluoromethylpyridin-2-yloxy)phenylmethyl]-oxime* (**8a**): White oil, yield 52%. ^1^H-NMR (CDCl_3_): δ 8.44 (s, 1H, Py-H), 8.08 (s, 1H, CH=N), 7.90 (d, *J* = 8.8 Hz, 1H, Py-H), 7.49 (d, *J* = 7.6 Hz, 2H, Ar-H and Py-H), 7.15 (d, *J* = 7.2 Hz, 2H, Ar-H), 7.01 (d, *J* = 8.8 Hz, 1H, Ar-H), 5.16 (s, 2H, CH_2_), 3.94 (s, 3H, OCH_3_), 3.62 (s, 3H, N-CH_3_), 2.28 (s, 3H, CH_3_); ^13^C-NMR (CDCl_3_): δ 165.8, 153.0, 152.8, 146.8, 145.5, 145.4, 141.6, 136.7, 136.6, 135.2, 130.0, 121.4, 111.3, 97.4, 75.4, 61.7, 33.6, 14.0. Anal. Calcd for C_20_H_19_F_3_N_4_O_3_: C 57.14; H 4.56; N 13.33. Found: C 57.28; H 4.39; N 13.16.

*1,3-Dimethyl-5-ethyloxy-1H-pyrazole-4-carbaldehyde-O-[4-(5-trifluoromethylpyridin-2-yloxy)phenylmethyl]-oxime* (**8b**). White solid, yield 55%, mp 48–50 °C. ^1^H-NMR (CDCl_3_): δ 8.42 (s, 1H, Py-H), 8.04 (s, 1H, CH=N), 7.88 (d, *J* = 8.8 Hz, 1H, Py-H), 7.47 (d, *J* = 7.2 Hz, 2H, Ar-H and Py-H), 7.14 (d, *J* = 6.8 Hz, 2H, Ar-H), 6.99 (d, *J* = 8.4 Hz, 1H, Ar-H), 5.14 (s, 2H, CH_2_), 4.16 (q, *J* = 6.8 Hz, 2H, CH_2_), 3.60 (s, 3H, N-CH_3_), 2.26 (s, 3H, CH_3_), 1.32 (t, *J* = 6.0 Hz, 3H, CH_3_); ^13^C-NMR (CDCl_3_): δ 165.8, 152.8, 152.1, 146.8, 145.4, 145.3, 141.7, 136.7, 136.6, 135.3, 130.0, 121.4, 111.3, 97.8, 75.3, 70.6, 33.6, 15.3, 14.0. Anal. Calcd for C_21_H_21_F_3_N_4_O_3_: C 58.06; H 4.87; N 12.90. Found: C 58.23; H 4.69; N 12.76.

*1,3-Dimethyl-5-tert-butyloxy-1H-pyrazole-4-carbaldehyde-O-[4-(5-trifluoromethylpyridin-2-yloxy)phenylmethyl]-oxime* (**8c**). White solid, yield 50%, mp 64–65 °C. ^1^H-NMR (CDCl_3_): δ 8.45 (s, 1H, Py-H), 8.01 (s, 1H, CH=N), 7.90 (d, *J* = 8.4 Hz, 1H, Py-H), 7.49 (d, *J* = 8.0 Hz, 2H, Ar-H and Py-H), 7.15 (d, *J* = 7.2 Hz, 2H, Ar-H), 7.01 (d, *J* = 8.8 Hz, 1H, Ar-H), 5.15 (s, 2H, CH_2_), 3.62 (s, 3H, N-CH_3_), 2.35 (s, 3H, CH_3_), 1.38 (s, 9H, *t*-C_4_H_9_); ^13^C-NMR (CDCl_3_): δ 165.8, 152.7, 150.1, 146.6, 145.5, 145.4, 143.0, 136.7, 135.3, 130.0, 125.1, 121.3, 111.3, 101.6, 85.4, 75.3, 34.6, 29.0, 15.2. Anal. Calcd for C_23_H_25_F_3_N_4_O_3_: C 59.73; H 5.45; N 12.11. Found: C 59.62; H 5.58; N 12.30.

*1,3-Dimethyl-5-(2-fluorophenoxy)-1H-pyrazole-4-carbaldehyde-O-[4-(5-trifluoromethylpyridin-2-yloxy)phenylmethyl]-oxime* (**8d**). Yellow solid, yield 48%, mp 56–58 °C. ^1^H-NMR (CDCl_3_): δ 8.46 (s, 1H, Py-H), 7.91 (d, *J* = 8.4 Hz, 1H, Py-H), 7.84 (s, 1H, CH=N), 7.37 (d, *J* = 6.8 Hz, 2H, Ar-H and Py-H), 7.01–7.22 (m, 6H, ArH), 6.78–6.82 (m, 1H, ArH), 5.01 (s, 2H, CH_2_), 3.68 (s, 3H, N-CH_3_), 2.38 (s, 3H, CH_3_). ^13^C-NMR (CDCl_3_): δ 165.8, 153.2, 152.8, 150.8, 147.3, 147.0, 145.5, 145.4, 144.3, 144.2, 140.4, 136.7, 135.0, 130.0, 124.6, 124.5, 121.3, 117.2, 117.1, 116.8, 111.3, 99.9, 75.5, 34.2, 14.5. Anal. Calcd for C_25_H_20_F_4_N_4_O_3_: C 60.00; H 4.03; N 11.20. Found: C 60.19; H 3.85; N 11.02.

*1,3-Dimethyl-5-(3-fluorophenoxy)-1H-pyrazole-4-carbaldehyde-O-[4-(5-trifluoromethylpyridin-2-yloxy)phenylmethyl]-oxime* (**8e**). White oil, yield 51%. ^1^H-NMR (CDCl_3_): δ 8.45 (s, 1H, Py-H), 7.91 (d, *J* = 8.8 Hz, 1H, Py-H), 7.86 (s, 1H, CH=N), 7.37 (d, *J* = 7.2 Hz, 2H, Ar-H and Py-H), 7.25–7.31 (m, 1H, ArH), 7.11 (d, *J* = 6.8 Hz, 2H, Ar-H), 7.01 (d, *J* = 8.4 Hz, 1H, Ar-H), 6.65–6.85 (m, 3H, ArH), 5.03 (s, 2H, CH_2_), 3.63 (s, 3H, N-CH_3_), 2.40 (s, 3H, CH_3_). ^13^C-NMR (CDCl_3_): δ 165.8, 164.8, 162.3, 157.6, 152.8, 147.0, 145.5, 140.5, 136.7, 134.9, 130.9, 130.8, 130.0, 125.1, 121.3, 111.3, 110.9, 110.8, 110.6, 103.7, 103.4, 100.4, 75.5, 34.2, 14.6. Anal. Calcd for C_25_H_20_F_4_N_4_O_3_: C 60.00; H 4.03; N 11.20. Found: C 59.85; H 4.21; N 11.39.

*1,3-Dimethyl-5-(4-fluorophenoxy)-1H-pyrazole-4-carbaldehyde-O-[4-(5-trifluoromethylpyridin-2-yloxy)phenylmethyl]-oxime* (**8f**). White solid, yield 56%, mp 46–48 °C. ^1^H-NMR (CDCl_3_): δ 8.44 (s, 1H, Py-H), 7.90 (d, *J* = 8.4 Hz, 1H, Py-H), 7.83 (s, 1H, CH=N), 7.36 (d, *J* = 8.0 Hz, 2H, Ar-H and Py-H), 7.11 (d, *J* = 8.4 Hz, 2H, Ar-H), 6.98–7.02 (m, 3H, ArH), 6.86–6,88 (m, 2H, ArH), 5.02 (s, 2H, CH_2_), 3.62 (s, 3H, N-CH_3_), 2.38 (s, 3H, CH_3_). ^13^C-NMR (CDCl_3_): δ 165.8, 160.0, 157.5, 152.8, 152.7, 147.7, 147.0, 145.5, 145.4, 140.6, 136.7, 135.0, 130.0, 125.1, 121.3, 120.0, 116.6, 116.5, 116.4, 111.4, 100.1, 75.4, 34.2, 14.6. Anal. Calcd for C_25_H_20_F_4_N_4_O_3_: C 60.00; H 4.03; N 11.20. Found: C 60.17; H 3.89; N 11.08.

*1,3-Dimethyl-5-(2-chlorophenoxy)-1H-pyrazole-4-carbaldehyde-O-[4-(5-trifluoromethylpyridin-2-yloxy)phenylmethyl]-oxime* (**8g**). White solid, yield 53%, mp 46–48 °C. ^1^H-NMR (CDCl_3_): δ 8.46 (s, 1H, Py-H), 7.91 (d, *J* = 8.8 Hz, 1H, Py-H), 7.82 (s, 1H, CH=N), 7.46 (d, *J* = 7.6 Hz, 1H, Ar-H), 7.36 (d, *J* = 7.6 Hz, 2H, Ar-H and Py-H), 7.05–7.19 (m, 4H, ArH), 7.01 (d, *J* = 8.8 Hz, 1H, Ar-H), 6.71 (d, *J* = 8.0 Hz, 1H, Ar-H), 5.01 (s, 2H, CH_2_), 3.66 (s, 3H, N-CH_3_), 2.38 (s, 3H, CH_3_). ^13^C-NMR (CDCl_3_): δ 165.8, 152.8, 152.2, 147.1, 147.0, 145.6, 145.5, 145.4, 140.3, 136.7, 134.9, 131.0, 130.1, 128.0, 124.6, 122.8, 121.3, 115.6, 111.3, 100.2, 75.5, 34.2, 14.5. Anal. Calcd for C_25_H_20_ClF_3_N_4_O_3_: C 58.09; H 3.90; N 10.84. Found: C 58.22; H 3.78; N 10.65.

*1,3-Dimethyl-5-(3-chlorophenoxy)-1H-pyrazole-4-carbaldehyde-O-[4-(5-trifluoromethylpyridin-2-yloxy)phenylmethyl]-oxime* (**8h**). White oil, yield 55%. ^1^H-NMR (CDCl_3_): δ 8.46 (s, 1H, Py-H), 7.91 (d, *J* = 8.4 Hz, 1H, Py-H), 7.85 (s, 1H, CH=N), 7.36 (d, *J* = 7.6 Hz, 2H, Ar-H and Py-H), 7.23–7.27 (m, 1H, ArH), 7.01–7.13 (m, 4H, ArH), 6.93 (s, 1H, Ar-H), 6.80 (d, *J* = 8.4 Hz, 1H, Ar-H), 5.02 (s, 2H, CH_2_), 3.63 (s, 3H, N-CH_3_), 2.39 (s, 3H, CH_3_). ^13^C-NMR (CDCl_3_): δ 165.8, 157.2, 152.8, 147.1, 146.9, 145.5, 145.4, 140.4, 136.7, 135.5, 134.9, 130.8, 130.1, 125.1, 124.0, 121.3, 116.0, 113.6, 111.3, 100.4, 75.5, 34.3, 14.5. Anal. Calcd for C_25_H_20_ClF_3_N_4_O_3_: C 58.09; H 3.90; N 10.84. Found: C 58.18; H 3.81; N 10.72.

*1,3-Dimethyl-5-(4-chlorophenoxy)-1H-pyrazole-4-carbaldehyde-O-[4-(5-trifluoromethylpyridin-2-yloxy)phenylmethyl]-oxime* (**8i**). White solid, yield 58%, mp 67–69 °C. ^1^H-NMR (CDCl_3_): δ 8.46 (s, 1H, Py-H), 7.91 (d, *J* = 8.4 Hz, 1H, Py-H), 7.83 (s, 1H, CH=N), 7.35 (d, *J* = 8.0 Hz, 2H, Ar-H and Py-H), 7.29 (d, *J* = 8.4 Hz, 2H, Ar-H), 7.12 (d, *J* = 7.6 Hz, 2H, Ar-H), 7.03 (d, *J* = 8.4 Hz, 1H, Ar-H), 6.85 (d, *J* = 8.4 Hz, 2H, Ar-H), 5.02 (s, 2H, CH_2_), 3.62 (s, 3H, N-CH_3_), 2.38 (s, 3H, CH_3_). ^13^C-NMR (CDCl_3_): δ 165.8, 155.2, 152.8, 147.2, 147.0, 145.5, 145.4, 140.5, 136.7, 134.9, 130.0, 129.9, 128.7, 121.3, 119.2, 116.6, 111.4, 100.3, 75.5, 34.2, 14.5. Anal. Calcd for C_25_H_20_ClF_3_N_4_O_3_: C 58.09; H 3.90; N 10.84. Found: C 57.93; H 4.06; N 10.98.

*1,3-Dimethyl-5-(2-bromophenoxy)-1H-pyrazole-4-carbaldehyde-O-[4-(5-trifluoromethylpyridin-2-yloxy)phenylmethyl]-oxime* (**8j**). White oil, yield 47%. ^1^H-NMR (CDCl_3_): δ 8.46 (s, 1H, Py-H), 7.91 (d, *J* = 8.4 Hz, 1H, Py-H), 7.82 (s, 1H, CH=N), 7.64 (d, *J* = 8.0 Hz, 1H, Ar-H), 7.36 (d, *J* = 7.6 Hz, 2H, Ar-H and Py-H), 7.10–7.24 (m, 3H, ArH), 7.02 (d, *J* = 8.0 Hz, 2H, Ar-H), 6.68 (d, *J* = 8.0 Hz, 1H, Ar-H), 5.01 (s, 2H, CH_2_), 3.66 (s, 3H, N-CH_3_), 2.39 (s, 3H, CH_3_). ^13^C-NMR (CDCl_3_): δ 165.8, 153.1, 152.8, 147.2, 147.0, 145.5, 140.3, 136.7, 134.0, 130.1, 128.8, 125.0, 121.3, 115.4, 111.4, 111.3, 100.2, 75.5, 34.3, 14.5. Anal. Calcd for C_25_H_20_BrF_3_N_4_O_3_: C 53.49; H 3.59; N 9.98. Found: C 53.62; H 3.40; N 9.80.

*1,3-Dimethyl-5-(3-bromophenoxy)-1H-pyrazole-4-carbaldehyde-O-[4-(5-trifluoromethylpyridin-2-yloxy)phenylmethyl]-oxime* (**8k**). White oil, yield 50%. ^1^H-NMR (CDCl_3_): δ 8.46 (s, 1H, Py-H), 7.91 (d, *J* = 8.4 Hz, 1H, Py-H), 7.85 (s, 1H, CH=N), 7.36 (d, *J* = 8.0 Hz, 2H, Ar-H and Py-H), 7.26 (d, *J* = 7.6 Hz, 1H, Ar-H), 7.09–7.21 (m, 4H, ArH), 7.02 (d, *J* = 8.4 Hz, 1H, Ar-H), 6.85 (d, *J* = 8.0 Hz, 1H, Ar-H), 5.02 (s, 2H, CH_2_), 3.63 (s, 3H, N-CH_3_), 2.39 (s, 3H, CH_3_). ^13^C-NMR (CDCl_3_): δ 165.8, 157.2, 152.8, 147.1, 146.8, 145.5, 145.4, 140.4, 136.7, 134.9, 131.1, 130.0, 126.9, 125.0, 123.2, 121.3, 118.9, 114.0, 111.3, 100.4, 75.5, 34.3, 14.5. Anal. Calcd for C_25_H_20_BrF_3_N_4_O_3_: C 53.49; H 3.59; N 9.98. Found: C 53.38; H 3.72; N 10.13.

*1,3-Dimethyl-5-(4-bromophenoxy)-1H-pyrazole-4-carbaldehyde-O-[4-(5-trifluoromethylpyridin-2-yloxy)phenylmethyl]-oxime* (**8l**). White solid, yield 53%, mp, 68–70 °C. ^1^H-NMR (CDCl_3_): δ 8.46 (s, 1H, Py-H), 7.92 (d, *J* = 8.8 Hz, 1H, Py-H), 7.84 (s, 1H, CH=N), 7.43 (d, *J* = 7.2 Hz, 2H, Ar-H), 7.35 (d, *J* = 7.2 Hz, 2H, Ar-H and Py-H), 7.12 (d, *J* = 7.2 Hz, 2H, Ar-H), 7.03 (d, *J* = 8.8 Hz, 1H, Ar-H), 6.80 (d, *J* = 7.2 Hz, 2H, Ar-H), 5.02 (s, 2H, CH_2_), 3.63 (s, 3H, N-CH_3_), 2.38 (s, 3H, CH_3_). ^13^C-NMR (CDCl_3_): δ 165.8, 155.8, 152.8, 147.1, 145.5, 145.4, 140.4, 136.7, 136.6, 134.9, 132.9, 130.1, 121.3, 117.1, 116.1, 111.4, 109.2, 100.3, 75.5, 34.2, 14.5. Anal. Calcd for C_25_H_20_BrF_3_N_4_O_3_: C 53.49; H 3.59; N 9.98. Found: C 53.32; H 3.76; N 10.09.

*1,3-Dimethyl-5-(4-iodophenoxy)-1H-pyrazole-4-carbaldehyde-O-[4-(5-trifluoromethylpyridin-2-yloxy)phenylmethyl]-oxime* (**8m**). White solid, yield 55%, mp 59–60 °C. ^1^H-NMR (CDCl_3_): δ 8.46 (s, 1H, Py-H), 7.91 (d, *J* = 8.8 Hz, 1H, Py-H), 7.83 (s, 1H, CH=N), 7.61 (d, *J* = 7.2 Hz, 2H, Ar-H), 7.35 (d, *J* = 7.6 Hz, 2H, Ar-H and Py-H), 7.12 (d, *J* = 6.8 Hz, 2H, Ar-H), 7.03 (d, *J* = 8.4 Hz, 1H, Ar-H), 6.69 (d, *J* = 7.2 Hz, 2H, Ar-H), 5.01 (s, 2H, CH_2_), 3.61 (s, 3H, N-CH_3_), 2.38 (s, 3H, CH_3_). ^13^C-NMR (CDCl_3_): δ 165.8, 156.6, 152.8, 147.0, 146.9, 145.5, 145.4, 140.4, 138.8, 136.7, 134.9, 130.1, 125.1, 121.3, 117.6, 111.4, 100.3, 86.4, 75.5, 34.2, 14.5. Anal. Calcd for C_25_H_20_F_3_IN_4_O_3_: C 49.36; H 3.31; N 9.21. Found: C 49.51; H 3.16; N 9.03.

*1,3-Dimethyl-5-phenoxy-1H-pyrazole-4-carbaldehyde-O-[4-(5-trifluoromethylpyridin-2-yloxy)phenylmethyl]-oxime* (**8n**). White solid, yield 56%, mp 45–46 °C. ^1^H-NMR (CDCl_3_): δ 8.46 (s, 1H, Py-H), 7.91 (d, *J* = 8.4 Hz, 1H, Py-H), 7.84 (s, 1H, CH=N), 7.34–7.39 (m, 4H, ArH and Py-H), 7.01–7.12 (m, 4H, ArH), 6.92 (d, *J* = 7.6 Hz, 2H, Ar-H), 5.03 (s, 2H, CH_2_), 3.63 (s, 3H, N-CH_3_), 2.40 (s, 3H, CH_3_). ^13^C-NMR (CDCl_3_): δ 165.8, 156.8, 152.8, 147.8, 146.9, 145.5, 145.4, 140.8, 136.7, 135.0, 130.1, 130.0, 125.1, 123.7, 121.3, 115.3, 111.3, 100.3, 75.5, 34.2, 14.8. Anal. Calcd for C_25_H_21_F_3_N_4_O_3_: C 62.24; H 4.39; N 11.61. Found: C 62.41; H 4.23; N 11.42.

*1,3-Dimethyl-5-(2-methoxyphenoxy)-1H-pyrazole-4-carbaldehyde-O-[4-(5-trifluoromethylpyridin-2-yloxy)phenylmethyl]-oxime* (**8o**). White solid, yield 52%, mp 55–57 °C. ^1^H-NMR (CDCl_3_): δ 8.45 (s, 1H, Py-H), 7.90 (d, *J* = 8.8 Hz, 1H, Py-H), 7.80 (s, 1H, CH=N), 7.38 (d, *J* = 8.0 Hz, 2H, Ar-H and Py-H), 7.00–7.12 (m, 5H, ArH), 6.71–6.88 (m, 2H, ArH), 5.03 (s, 2H, CH_2_), 3.91 (s, 3H, OCH_3_), 3.65 (s, 3H, N-CH_3_), 2.38 (s, 3H, CH_3_). ^13^C-NMR (CDCl_3_): δ 165.8, 152.7, 149.0, 148.5, 146.8, 145.8, 145.5, 145.4, 140.9, 136.7, 136.6, 135.1, 130.1, 124.6, 121.3, 121.0, 115.9, 112.8, 111.3, 99.6, 75.4, 56.1, 34.2, 14.9. Anal. Calcd for C_26_H_23_F_3_N_4_O_4_: C 60.93; H 4.52; N 10.93. Found: C 60.78; H 4.71; N 11.06.

*1,3-Dimethyl-5-(4-methoxyphenoxy)-1H-pyrazole-4-carbaldehyde-O-[4-(5-trifluoromethylpyridin-2-yloxy)phenylmethyl]-oxime* (**8p**). White solid, yield 63%, mp 76–78 °C. ^1^H-NMR (CDCl_3_): δ 8.45 (s, 1H, Py-H), 7.90 (d, *J* = 8.8 Hz, 1H, Py-H), 7.82 (s, 1H, CH=N), 7.39 (d, *J* = 8.4 Hz, 2H, Ar-H and Py-H), 7.11 (d, *J* = 8.4 Hz, 2H, Ar-H), 7.01 (d, *J* = 8.4 Hz, 1H, Ar-H), 6.85 (s, 4H, Ar-H), 5.04 (s, 2H, CH_2_), 3.77 (s, 3H, OCH_3_), 3.62 (s, 3H, N-CH_3_), 2.38 (s, 3H, CH_3_). ^13^C-NMR (CDCl_3_): δ 165.8, 155.8, 152.8, 150.7, 148.5, 146.9, 145.5, 145.4, 140.9, 136.7, 136.6, 135.0, 130.1, 121.3, 116.4, 114.9, 111.3, 99.9, 75.4, 55.7, 34.2, 14.8. Anal. Calcd for C_26_H_23_F_3_N_4_O_4_: C 60.93; H 4.52; N 10.93. Found: C 61.04; H 4.35; N 10.78.

*1,3-Dimethyl-5-(4-methylphenoxy)-1H-pyrazole-4-carbaldehyde-O-[4-(5-trifluoromethylpyridin-2-yloxy)phenylmethyl]-oxime* (**8q**). White solid, yield 61%, mp 52–53 °C. ^1^H-NMR (CDCl_3_): δ 8.46 (s, 1H, Py-H), 7.90 (d, *J* = 8.4 Hz, 1H, Py-H), 7.84 (s, 1H, CH=N), 7.39 (d, *J* = 7.6 Hz, 2H, Ar-H and Py-H), 7.12 (d, *J* = 7.2 Hz, 4H, Ar-H), 7.01 (d, *J* = 8.4 Hz, 1H, Ar-H), 6.81 (d, *J* = 7.6 Hz, 2H, Ar-H), 5.05 (s, 2H, CH_2_), 3.61 (s, 3H, N-CH_3_), 2.40 (s, 3H, CH_3_), 2.32 (s, 3H, CH_3_). ^13^C-NMR (CDCl_3_): δ 165.8, 154.8, 152.8, 148.1, 146.8, 145.5, 145.4, 140.9, 136.7, 136.6, 135.1, 133.1, 130.4, 130.1, 121.3, 115.1, 111.3, 100.1, 75.4, 34.2, 20.5, 14.9. Anal. Calcd for C_26_H_23_F_3_N_4_O_3_: C 62.90; H 4.67; N 11.28. Found: C 62.73; H 4.81; N 11.42.

*1,3-Dimethyl-5-(4-tert-butylphenoxy)-1H-pyrazole-4-carbaldehyde-O-[4-(5-trifluoromethylpyridin-2-yloxy)phenylmethyl]-oxime* (**8r**). White oil, yield 57%. ^1^H-NMR (CDCl_3_): δ 8.46 (s, 1H, Py-H), 7.91 (d, *J* = 8.8 Hz, 1H, Py-H), 7.85 (s, 1H, CH=N), 7.41 (d, *J* = 8.0 Hz, 2H, Ar-H and Py-H), 7.12 (d, *J* = 8.4 Hz, 2H, Ar-H), 7.02 (d, *J* = 8.8 Hz, 1H, Ar-H), 6.84 (d, *J* = 8.4 Hz, 2H, Ar-H), 5.05 (s, 2H, CH_2_), 3.62 (s, 3H, N-CH_3_), 2.41 (s, 3H, CH_3_), 1.32 (s, 9H, *t*-C_4_H_9_). ^13^C-NMR (CDCl_3_): δ 165.8, 154.6, 152.8, 148.1, 146.8, 145.5, 145.4, 141.0, 136.7, 136.6, 135.0, 130.1, 126.8, 121.3, 100.2, 75.4, 34.3, 34.2, 31.4, 14.9. Anal. Calcd for C_29_H_29_F_3_N_4_O_3_: C 64.67; H 5.43; N 10.40. Found: C 64.52; H 5.61; N 10.58.

*1,3-Dimethyl-5-(4-trifluoromethoxyphenoxy)-1H-pyrazole-4-carbaldehyde-O-[4-(5-trifluoromethylpyridin-2-yloxy)phenylmethyl]-oxime* (**8s**). White solid, yield 52%, mp 75–77 °C. ^1^H-NMR (CDCl_3_): δ 8.45 (s, 1H, Py-H), 7.91 (d, *J* = 8.4 Hz, 1H, Py-H), 7.85 (s, 1H, CH=N), 7.36 (d, *J* = 8.0 Hz, 2H, Ar-H and Py-H), 7.19 (d, *J* = 8.4 Hz, 2H, Ar-H), 7.11 (d, *J* = 8.0 Hz, 2H, Ar-H), 7.02 (d, *J* = 8.4 Hz, 1H, Ar-H), 6.92 (d, *J* = 8.8 Hz, 2H, Ar-H), 5.00 (s, 2H, CH_2_), 3.64 (s, 3H, N-CH_3_), 2.39 (s, 3H, CH_3_). ^13^C-NMR (CDCl_3_): δ 154.9, 152.8, 147.1, 145.5, 140.4, 136.7, 134.8, 130.0, 122.8, 121.3, 116.4, 111.4, 100.3, 75.5, 34.2, 14.5. Anal. Calcd for C_26_H_20_F_6_N_4_O_4_: C 55.13; H 3.56; N 9.89. Found: C 55.01; H 3.71; N 10.03.

*1,3-Dimethyl-5-(2,3-difluorophenoxy)-1H-pyrazole-4-carbaldehyde-O-[4-(5-trifluoromethylpyridin-2-yloxy)phenylmethyl]-oxime* (**8t**). White solid, yield 44%, mp 61–62 °C. ^1^H-NMR (CDCl_3_): δ 8.45 (s, 1H, Py-H), 7.91 (d, *J* = 8.8 Hz, 1H, Py-H), 7.85 (s, 1H, CH=N), 7.36 (d, *J* = 8.0 Hz, 2H, Ar-H and Py-H), 7.11 (d, *J* = 8.0 Hz, 2H, Ar-H), 7.02 (d, *J* = 8.4 Hz, 1H, Ar-H), 6.93–6.97 (m, 2H, ArH), 6.54 (d, *J* = 8.4 Hz, 1H, Ar-H), 5.00 (s, 2H, CH_2_), 3.68 (s, 3H, N-CH_3_), 2.36 (s, 3H, CH_3_). ^13^C-NMR (CDCl_3_): δ 165.8, 152.8, 147.1, 146.7, 145.5, 145.4, 140.1, 136.7, 136.6, 134.9, 129.9, 123.5, 123.4, 121.3, 112.3, 112.1, 111.6, 111.5, 111.3, 100.0, 75.5, 34.2, 14.3. Anal. Calcd for C_25_H_19_F_5_N_4_O_3_: C 57.92; H 3.69; N 10.81. Found: C 57.76; H 3.83; N 10.96.

*1,3-Dimethyl-5-(2,4-dichlorophenoxy)-1H-pyrazole-4-carbaldehyde-O-[4-(5-trifluoromethylpyridin-2-yloxy)phenylmethyl]-oxime* (**8u**). White solid, yield 52%, mp 109–111 °C. ^1^H-NMR (CDCl_3_): δ 8.45 (s, 1H, Py-H), 7.90 (d, *J* = 8.4 Hz, 1H, Py-H), 7.82 (s, 1H, CH=N), 7.46 (s, 1H, Ar-H), 7.33 (d, *J* = 8.4 Hz, 2H, Ar-H and Py-H), 7.00–7.12 (m, 4H, ArH), 6.63 (d, *J* = 8.8 Hz, 2H, Ar-H), 4.98 (s, 2H, CH_2_), 3.65 (s, 3H, N-CH_3_), 2.36 (s, 3H, CH_3_). ^13^C-NMR (CDCl_3_): δ 165.8, 152.8, 150.9, 147.1, 146.5, 145.5, 145.4, 140.1, 136.7, 134.9, 130.5, 130.0, 129.0, 127.9, 125.1, 123.6, 122.4, 121.3, 116.3, 111.4, 100.1, 75.5, 34.2, 14.2. Anal. Calcd for C_25_H_19_Cl_2_F_3_N_4_O_3_: C 54.46; H 3.47; N 10.16. Found: C 54.62; H 3.29; N 10.02.

*1,3-Dimethyl-5-(2,3-dimethylphenoxy)-1H-pyrazole-4-carbaldehyde-O-[4-(5-trifluoromethylpyridin-2-yloxy)phenylmethyl]-oxime* (**8v**). White oil, yield 49%. ^1^H-NMR (CDCl_3_): δ 8.46 (s, 1H, Py-H), 7.91 (d, *J* = 8.0 Hz, 1H, Py-H), 7.77 (s, 1H, CH=N), 7.38 (d, *J* = 8.0 Hz, 2H, Ar-H and Py-H), 7.12 (d, *J* = 8.0 Hz, 2H, Ar-H), 6.93–7.03 (m, 3H, ArH), 6.42 (d, *J* = 8.0 Hz, 1H, Ar-H), 5.04 (s, 2H, CH_2_), 3.62 (s, 3H, N-CH_3_), 2.41 (s, 3H, CH_3_), 2.35 (s, 3H, CH_3_), 2.32 (s, 3H, CH_3_). ^13^C-NMR (CDCl_3_): δ 165.8, 154.9, 152.8, 148.6, 146.9, 145.5, 141.0, 139.0, 136.7, 136.6, 135.1, 130.1, 126.2, 125.2, 121.3, 111.3, 111.2, 99.8, 75.4, 34.1, 20.0, 14.9, 11.8. Anal. Calcd for C_27_H_25_F_3_N_4_O_3_: C 63.52; H 4.94; N 10.97. Found: C 63.36; H 5.09; N 11.05.

*1,3-Dimethyl-5-(2,4-dimethylphenoxy)-1H-pyrazole-4-carbaldehyde-O-[4-(5-trifluoromethylpyridin-2-yloxy)phenylmethyl]-oxime* (**8w**). White oil, yield 53%. ^1^H-NMR (CDCl_3_): δ 8.46 (s, 1H, Py-H), 7.91 (d, *J* = 8.4 Hz, 1H, Py-H), 7.76 (s, 1H, CH=N), 7.38 (d, *J* = 8.0 Hz, 2H, Ar-H and Py-H), 7.11 (d, *J* = 7.6 Hz, 2H, Ar-H), 6.88–7.05 (m, 3H, ArH), 6.45 (d, *J* = 8.4 Hz, 1H, Ar-H), 5.04 (s, 2H, CH_2_), 3.62 (s, 3H, N-CH_3_), 2.40 (s, 3H, CH_3_), 2.36 (s, 3H, CH_3_), 2.29 (s, 3H, CH_3_). ^13^C-NMR (CDCl_3_): δ 165.8, 152.9, 152.7, 148.6, 146.9, 145.5, 141.0, 136.7, 135.1, 133.0, 132.2, 130.1, 127.5, 126.2, 125.1, 122.4, 121.7, 121.3, 113.4, 99.7, 75.4, 34.1, 20.5, 16.0, 14.9. Anal. Calcd for C_27_H_25_F_3_N_4_O_3_: C 63.52; H 4.94; N 10.97. Found: C 63.69; H 4.78; N 10.82.

### 3.2. Biological Tests

#### 3.2.1. Bioassay Methods

All bioassays were performed on representative test organisms reared in the laboratory. The bioassay was repeated in triplicate at 25 ± 1 °C. Assessments were made on a dead/alive basis, and mortality rates were corrected using Abbott’s formula. For comparative purposes, the controls Fenpyroximate, Pyridalyl and Imidacloprid were evaluated under the same conditions.

#### 3.2.2. Acaricidal Activity against *Tetranychus cinnabarinus*

The acaricidal activities against *Tetranychus cinnabarinus* of the designed compounds were evaluated using the reported procedure [27]. Sieva bean plants with primary leaves expanded to 10 cm were selected and cut back to one plant per pot. A small piece was cut from a leaf taken from the main colony and placed on each leaf of the test plants. This was done about 2 h before treatment to allow the mites to move over to the test plant and to lay eggs. The size of the piece was varied to obtain about 60–100 mites per leaf. At the time of the treatment, the piece of leaf used to transfer the mites was removed and discarded. The mite-infested plants were dipped in the test formulation for 3 s with agitation and set in the hood to dry. Plants were kept for 48 h before the numbers of live and dead adults were counted. Each experiment for one compound was triplicated.

#### 3.2.3. Insecticidal Activity against *Plutella xylostella*

The insecticidal activities of the title compounds against *Plutella xylostella* were evaluated using the leaf disk assay [28]. First, a solution of each test sample in *N*,*N*-dimethylformamide at a concentration of 200 μg/mL was prepared and then diluted to the required concentration with water. Cabbage leaves were dipped into the obtained solutions for 2–3 s. After air-drying, the soaked leaves were put into a 10-cm-long tube, inoculated with second *Plutella xylostella* larva. Covered with gauze and then kept in a room for normal cultivation. Mortality was assessed 48 h after treatment. Each experiment for one compound was triplicated.

#### 3.2.4. Insecticidal Activity against *Aphis craccivora*

Insecticidal activities of the target compounds were tested against *Aphis craccivora* by foliar application [29]. About 60 aphids were transferred to the shoot with 3–5 fresh leaves of horsebean. The shoot with aphids was cut and dipped into a required solution from 200 μg/mL to 100 μg/mL of the tested compound for 2 s. After removing extra solutions on the leaf, the aphids were raised in the shoot at 25 °C and 85% relative humidity for 48 h. Each experiment for one compound was triplicated.

## 4. Conclusions

In summary, 23 pyrazole oxime compounds bearing a 5-trifluoromethyl pyridyl subunit were synthesized. A preliminary evaluation of the acaricidal and insecticidal activities of the designed compounds was conducted. Most of them exhibited obvious acaricidal activity against *T. cinnabarinus* at a concentration of 200 μg/mL, and some derivatives such as compounds **8e**, **8f**, **8l**, **8m**, **8n**, **8p**, and **8q** still possessed excellent acaricidal activity against *T. cinnabarinus* under the concentration of 10 μg/mL. Additionally, some compounds showed potent insecticidal activities against *P. xylostella* and *A. craccivora* at a concentration of 200 μg/mL. Notably, compounds **8e** and **8l** were more active against *P. xylostella* than other compounds, even when the concentration was decreased to 50 μg/mL. Among these compounds, compounds **8e**, **8i**, and **8n** showed broad spectrum biological activities; they displayed potential insecticidal activity against *P. xylostella* and *A. craccivora* and beyond satisfactory acaricidal activity against *T. cinnabarinus*. Further investigations on the structural optimization and bioactivities of these pyrazole oximes are currently in progress.

## Figures and Tables

**Table 1:** Acaricidal activities of compounds **8a**–**8w** (mortality, %).

Compd.	R	*Tetranychus* *cinnabarinus*			
		200 μg/mL	100 μg/mL	50 μg/mL	10 μg/mL
**8a**	Me	0	— ^b^	—	—
**8b**	Et	0	—	—	—
**8c**	*t*-Bu	0	—	—	—
**8d**	2-FC_6_H_4_	100.00 ± 0.00	90.32 ± 1.38	70.43 ± 0.82	0
**8e**	3-FC_6_H_4_	100.00 ± 0.00	100.00 ± 0.00	100.00 ± 0.00	100.00 ± 0.00
**8f**	4-FC_6_H_4_	100.00 ± 0.00	100.00 ± 0.00	100.00 ± 0.00	100.00 ± 0.00
**8g**	2-ClC_6_H_4_	70.28 ± 1.33 ^a^	0	—	—
**8h**	3-ClC_6_H_4_	100.00 ± 0.00	30.39 ± 0.67	—	—
**8i**	4-ClC_6_H_4_	100.00 ± 0.00	100.00 ± 0.00	100.00 ± 0.00	50.49 ± 1.78
**8j**	2-BrC_6_H_4_	80.16 ± 0.38	30.46 ± 1.52	0	—
**8k**	3-BrC_6_H_4_	70.33 ± 1.22	0	—	—
**8l**	4-BrC_6_H_4_	100.00 ± 0.00	100.00 ± 0.00	100.00 ± 0.00	100.00 ± 0.00
**8m**	4-IC_6_H_4_	100.00 ± 0.00	100.00 ± 0.00	100.00 ± 0.00	100.00 ± 0.00
**8n**	C_6_H_5_	100.00 ± 0.00	100.00 ± 0.00	100.00 ± 0.00	100.00 ± 0.00
**8o**	2-OMeC_6_H_4_	0	—	—	—
**8p**	4-OMeC_6_H_4_	100.00 ± 0.00	100.00 ± 0.00	100.00 ± 0.00	100.00 ± 0.00
**8q**	4-MeC_6_H_4_	100.00 ± 0.00	100.00 ± 0.00	100.00 ± 0.00	100.00 ± 0.00
**8r**	4-*t*-BuC_6_H_4_	0	—	—	—
**8s**	4-OCF_3_C_6_H_4_	100.00± 0.00	100.00 ± 0.00	100.00 ± 0.00	95.29 ± 1.45
**8t**	2,3-F_2_C_6_H_3_	100.00 ± 0.00	100.00 ± 0.00	80.42 ± 1.56	—
**8u**	2,4-Cl_2_C_6_H_3_	80.17 ± 0.82	50.23 ± 1.43	0	—
**8v**	2,3-Me_2_C_6_H_3_	0	—	—	—
**8w**	2,4-Me_2_C_6_H_3_	100.00 ± 0.00	100.00 ± 0.00	60.35 ± 1.45	—
Fenpyroximate		100.00 ± 0.00	100.00 ± 0.00	100.00 ± 0.00	100.00 ± 0.00

^a^ Each value represents the mean ± standard error of three replications; ^b^ “—” refers to “not tested”.

**Table 2:** Insecticidal activities of compounds **8a**–**8w** (mortality, %).

Compd.	R	*Plutella xylostella*		*Aphis* *craccivora*	
		200 μg/mL	50 μg/mL	200 μg/mL	100 μg/mL
**8a**	Me	100.00 ± 0.00	0	30.26 ± 1.56	—
**8b**	Et	0	— ^b^	0	—
**8c**	*t*-Bu	0	—	0	—
**8d**	2-FC_6_H_4_	0	—	0	—
**8e**	3-FC_6_H_4_	100.00 ± 0.00	86.42 ± 0.88	100.00 ± 0.00	60.18 ± 1.33
**8f**	4-FC_6_H_4_	100.00 ± 0.00	43.36 ± 1.07	60.29 ± 1.52	30.41 ± 0.71
**8g**	2-ClC_6_H_4_	0	—	0	—
**8h**	3-ClC_6_H_4_	30.29 ± 1.55 ^a^	0	85.33 ± 1.25	50.27 ± 1.06
**8i**	4-ClC_6_H_4_	100.00 ± 0.00	71.23 ± 0.95	95.28 ± 1.32	60.26 ± 1.69
**8j**	2-BrC_6_H_4_	0	—	20.41	—
**8k**	3-BrC_6_H_4_	57.35 ± 0.54	29.16 ± 1.86	0	—
**8l**	4-BrC_6_H_4_	100.00 ± 0.00	100.00 ± 0.00	50.37 ± 1.32	0
**8m**	4-IC_6_H_4_	100.00 ± 0.00	71.39 ± 1.53	0	—
**8n**	C_6_H_5_	100.00 ± 0.00	71.18 ± 0.65	70.25 ± 1.63	50.43 ± 1.38
**8o**	2-OMeC_6_H_4_	0	—	0	—
**8p**	4-OMeC_6_H_4_	100.00 ± 0.00	43.41 ± 1.71	70.32 ± 1.95	20.17 ± 0.71
**8q**	4-MeC_6_H_4_	100.00 ± 0.00	71.22 ± 1.24	0	—
**8r**	4-*t*-BuC_6_H_4_	0	—	100.00 ± 0.00	40.09 ± 0.36
**8s**	4-OCF_3_C_6_H_4_	57.27 ± 0.83	43.35 ± 1.65	—	—
**8t**	2,3-F_2_C_6_H_3_	43.09 ± 1.26	0	—	—
**8u**	2,4-Cl_2_C_6_H_3_	43.25 ± 1.07	0	—	—
**8v**	2,3-Me_2_C_6_H_3_	0	—	—	—
**8w**	2,4-Me_2_C_6_H_3_	43.18 ± 0.92	0	—	—
Pyridalyl		100.00 ± 0.00	100.00 ± 0.00	—	—
Imidacloprid		—	—	100.00 ± 0.00	100.00 ± 0.00

^a^ Each value represents the mean ± standard error of three replications; ^b^ “—” refers to “not tested”.
